# In Vitro Susceptibility of Clinical Isolates to Ceftriaxone Alone and Ceftriaxone in Combination With Sulbactam or Tazobactam: A Comparative Study of Broad-Spectrum β-Lactam Antibiotics in India

**DOI:** 10.7759/cureus.46014

**Published:** 2023-09-26

**Authors:** Sonali Sanghavi, Ujjala Ghoshal, Sumon Poddar, Meenakshi Satpute, Chinmoy Sahu, Dattatray Pawar, Akhilesh Sharma, Pooja H Vaidya

**Affiliations:** 1 Microbiology, KEM Hospital, Pune, IND; 2 Microbiology, Sanjay Gandhi Postgraduate Institute of Medical Sciences, Lucknow, IND; 3 Microbiology, Institute of Child Health, Kolkata, IND; 4 Medical Affairs, Alkem Laboratories Ltd., Mumbai, IND

**Keywords:** tazobactam, susceptibility, sulbactam, minimum inhibitory concentration (mic), efficacy ratio (er), ceftriaxone

## Abstract

Background

This study was designed to evaluate the current in vitro susceptibility of clinical isolates to broad-spectrum β-lactam antibiotics.

Methodology

Bacterial isolates, cultured from 180 non-repetitive clinical samples between April and November 2022 at three hospitals in India, were used to evaluate the minimum inhibitory concentration (MIC) of broad-spectrum β-lactam antibiotics using the Epsilometer test (E-test) method. Test antibiotics were ceftriaxone and ceftriaxone in combination with β-lactamase inhibitors (BLIs) sulbactam and tazobactam. Comparator antibiotics included amoxicillin + BLI clavulanic acid, piperacillin + tazobactam, cefotaxime, and cefepime. The MIC values obtained were used to assess the susceptibility of the isolates and to compute the efficacy ratios (ERs) of the antibiotics.

Results

Among the 180 clinical isolates, ~89% were gram-negative bacteria, the most prevalent ones being *Escherichia coli *and* Klebsiella pneumoniae*. Of the gram-negative isolates, ~37% were susceptible/intermediately susceptible to ceftriaxone, and ~29% were susceptible to ceftriaxone + BLIs. The test antibiotics had ER >10 against 85%-95% *E. coli *isolates, whereas comparator antibiotics had ER >10 against 31%-68% isolates. The differences between the test antibiotics and piperacillin + tazobactam or cefotaxime were statistically significant. Ceftriaxone, ceftriaxone + sulbactam, and ceftriaxone + tazobactam had ER >10 against 78%, 100%, and 90% of *K. pneumoniae* isolates, while the corresponding percentages for cefotaxime, piperacillin + tazobactam, and cefepime were 100%, 64%, and 80%, respectively. The difference between ceftriaxone + BLIs and piperacillin + tazobactam was statistically significant. Ceftriaxone + BLIs had ER >10 against all *E. coli *isolates producing extended-spectrum β-lactamases (ESBLs); the percentage of isolates was significantly higher than that for piperacillin + tazobactam. Ceftriaxone + tazobactam had ER >10 against all ESBL-producing *K. pneumoniae* isolates; ceftriaxone and ceftriaxone + sulbactam had ER ranging 6-10.

Conclusions

Ceftriaxone and ceftriaxone in combination with sulbactam and tazobactam are promising antibiotics to explore against prevalent infectious microorganisms such as *E. coli* and *K. pneumoniae*. Ceftriaxone + tazobactam also holds promise against ESBL-producing variants.

## Introduction

Antibiotics have revolutionized modern medicine, and within a century of their discovery, extended the average human lifespan. However, this armamentarium that potentiated mankind’s victory against infectious organisms currently faces the challenge of withstanding the pace of antimicrobial resistance (AMR). Globally, 4.95 million deaths were estimated to be associated with bacterial AMR in 2019 [1]. AMR is especially of concern in India due to the easy availability of antibiotics (often without prescription) and increased consumption of antibiotics (often without medical supervision). This is alarming as the burden of infectious diseases in India is among the highest in the world [2].

Cephalosporins are a class of β-lactam antibiotics which are highly effective bactericidal agents. These disrupt the cross-linking of peptidoglycan chains and irreversibly inhibit bacterial cell wall synthesis. The cephalosporin antibiotics developed to date are usually categorized into five major generations based on the chronology of their discovery and their spectrum of activity. Overall, lower-generation cephalosporins have more gram-positive activity while higher-generation cephalosporins are more potent against gram-negative bacteria, although there are exceptions. Higher-generation cephalosporins were designed to target resistance mechanisms in bacteria or to have increased stability to such mechanisms. Thus, with an increase in the generation, the spectrum of activity of a cephalosporin antibiotic is broadened. Ceftriaxone is a third-generation cephalosporin with a broad spectrum of activity against gram-negative and most gram-positive microorganisms [3].

Bacteria develop resistance to β-lactam antibiotics by the production of β-lactamase enzyme. To circumvent this, many β-lactam antibiotics are administered concomitantly with β-lactamase inhibitors (BLIs) [4]. β-lactamases are divided into four classes: the active-site serine β-lactamases (SBLs; classes A, C, and D) and the zinc-dependent metallo-β-lactamases (MBLs; class B). The indiscriminate use of β-lactam antibiotics has led to the emergence of a variety of mutated forms of β-lactamases, one of which is the extended-spectrum β-lactamases (ESBLs) among the SBLs. ESBL- and MBL-producing bacteria pose a significant threat to the treatment of infections [5]. Therefore, as part of our study, we categorized the clinical isolates into ESBL and/or MBL producers and non-producers and analyzed them separately.

Considering the trend of AMR, it is imperative to re-evaluate microbial susceptibility. This study aimed to define current susceptibility/resistance patterns of gram‑negative and gram-positive clinical isolates to ceftriaxone alone and ceftriaxone in combination with BLIs, sulbactam and tazobactam. The study was designed to aid clinical decision-making regarding the most appropriate antibiotic choice. The primary endpoint of the study was the minimum inhibitory concentration (MIC) values of ceftriaxone alone and ceftriaxone in combination with sulbactam and tazobactam. MIC, as the expanded name suggests, is the minimum concentration of antibiotic at which the growth of a microorganism is inhibited/impaired.

An essential requisite of such susceptibility testing is the *clinical breakpoint* that defines the susceptibility and resistance of a microorganism. Determination of clinical breakpoint is dependent on the availability of in vitro microbiological data (e.g., MIC), pharmacokinetic/pharmacodynamic data from animal and human studies, and data of clinical/bacteriological outcomes from clinical studies. Isolates in the *susceptible* category are inhibited by the usually achievable concentrations of antimicrobial agents, while those in the *resistant* category are not. The *intermediate* isolates reside between these two categories. Thus, the clinical breakpoint of an antibacterial agent against an infectious organism informs clinicians on the likelihood of the antibacterial agent being clinically useful in the treatment of diseases caused by the aforementioned organism [6]. As the clinical breakpoint is different for each antibiotic-microorganism pair, the direct comparison of MICs of antibiotics is not worthwhile. Therefore, we evaluated efficacy ratios (ERs) to compare the degree of susceptibility of clinical isolates to antibiotics [7]. ER is a ratio of susceptible MIC breakpoint to the MIC of clinical isolate; it is defined for each pair of antibiotic and bacterial species. ER provides additional information on how the observed MIC of an antibiotic compares to its known breakpoint. A higher ER may correlate with a higher therapeutic efficacy.

## Materials and methods

This study on ceftriaxone in vitro susceptibility assessment was conducted in accordance with the principles of the Declaration of Helsinki and Good Clinical Practices guidelines, as mentioned in New Drugs and Clinical Trials Rules 2019, issued by the Central Drugs Standard Control Organization, Ministry of Health, Government of India. This study was conducted in compliance with the approved clinical study protocol. Approval from the Institutional Ethics Committee of each study site was obtained before study initiation. The trial was registered with the Clinical Trials Registry of India on April 20, 2022 (reference number: CTRI/2022/04/041997).

The study was conducted between April and November 2022 at three hospitals in India, namely, Sanjay Gandhi Postgraduate Institute of Medical Sciences (SGPGI, Lucknow), KEM Hospital Research Centre (KEM, Pune), and Institute of Child Health (ICH, Kolkata). Bacterial isolates were cultured from a total of 180 non-repetitive clinical samples, including blood, pus, wound swab, urine, sputum, ascitic/peritoneal fluid, cyst fluid, bronchoalveolar lavage (BAL) fluid, etc. These clinical isolates were incubated in the presence of Ezy MIC™ strips (HiMedia Laboratories Pvt. Limited, Maharashtra, India) impregnated with a predefined concentration gradient of antibiotics. Test antibiotics included ceftriaxone, ceftriaxone + sulbactam, and ceftriaxone + tazobactam. Comparator antibiotics used in the study were semi-synthetic penicillin amoxicillin in combination with BLI clavulanic acid, ureidopenicillin piperacillin in combination with tazobactam, third‑generation cephalosporin cefotaxime, and a fourth-generation cephalosporin cefepime. MIC evaluation was performed using the Epsilometer test (E-test) according to the manufacturer’s instructions.

All statistical methods were based on the International Conference on Harmonization E9 document Statistical Principles for Clinical Trials. The number and percentage of clinical isolates were compared to interpret susceptibility to antibiotics. Isolates were classified as susceptible, intermediately susceptible, and resistant based on their known susceptibility ranges according to the information provided on the strips. As clinical breakpoints and susceptibility ranges are not available for ceftriaxone combinations at the Clinical and Laboratory Standards Institute and European Committee on Antimicrobial Susceptibility Testing, we decided to use the values available for ceftriaxone as a proxy for ceftriaxone + sulbactam and ceftriaxone + tazobactam combinations. ER was evaluated as the ratio of susceptible MIC breakpoint to the MIC of clinical isolate. Two-proportion z-test was used to compare the proportion of clinical isolates corresponding to different ER ranges of each test antibiotic to that of every other antibiotic used in the study.

## Results

Among the 180 clinical isolates, 160 (~89%) were gram-negative bacteria while only 20 (~11%) were gram-positive bacteria. The most commonly found gram-negative bacteria were *Escherichia coli* (70 isolates) and *Klebsiella pneumoniae* (37 isolates), two of the six leading causative pathogens identified in AMR-associated fatalities [1]. Other gram-negative bacteria detected were *Pseudomonas aeruginosa* (30 isolates), *Salmonella typhi* (five isolates), *Enterobacter aerogenes* (four isolates), *Proteus mirabilis* (four isolates), *Enterobacter cloacae* (three isolates), *Acinetobacter baumannii* (two isolates), and *Salmonella enterica*, *Citrobacter koseri*, *Pseudomonas putida*, *Ralstonia insidiosa*, and *Serratia marcescens* (one isolate, each). *Staphylococcus aureus* (19 isolates) and *Enterococcus faecalis* (one isolate) were the only gram‑positive bacteria isolated.

**Figure 1:**
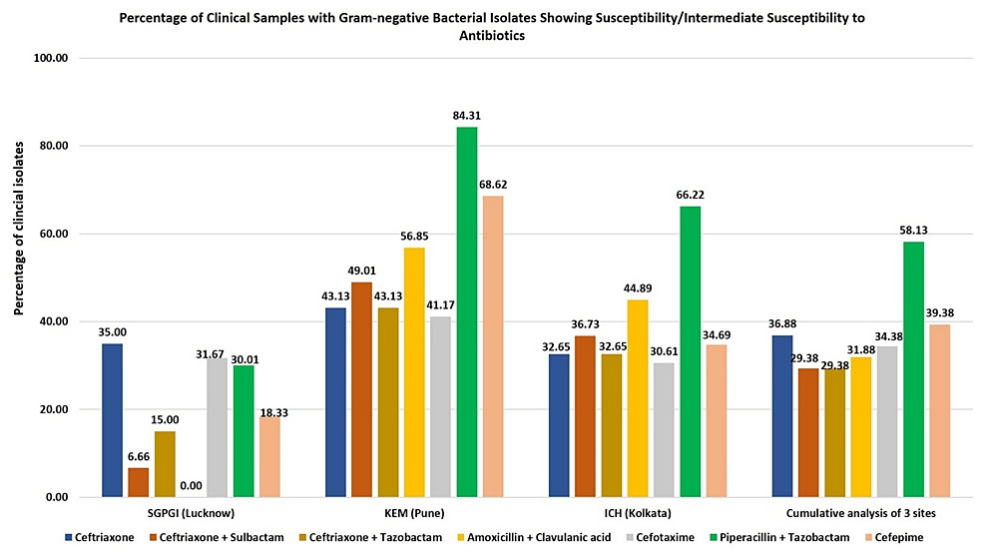
Percentage of gram-negative isolates showing susceptibility/intermediate susceptibility to test antibiotics.

Based on the MIC values obtained using E-test, we performed susceptibility analysis for these isolates. Cumulative analysis of all three study sites showed that ~37% of gram-negative bacterial isolates were susceptible/intermediately susceptible to ceftriaxone, and the corresponding percentage for ceftriaxone in combination with any of the BLIs was ~29%. Varying susceptibility rates were observed at individual sites. The rates of susceptibility/intermediate susceptibility to ceftriaxone were 43%, 35%, and 33% at KEM (Pune), SGPGI (Lucknow), and ICH (Kolkata), respectively. The corresponding values for ceftriaxone in combination with BLIs were 43%-49% at KEM (Pune) and 33%-37% at ICH (Kolkata), which were higher than the 6%-15% observed at SGPGI (Lucknow). Overall, the rate of susceptibility/intermediate susceptibility to ceftriaxone (35%) was higher than that of all other antibiotics tested at SGPGI (Lucknow) (Figure 1).

We performed a similar analysis for the comparator antibiotics. While the rate of susceptibility/intermediate susceptibility of gram-negative bacterial isolates to amoxicillin + clavulanic acid was high at KEM (Pune) and ICH (Kolkata) (57% and 45%, respectively), all gram-negative isolates at SGPGI (Lucknow) were resistant to this antibiotic. Similar was the case for piperacillin + tazobactam: rates of susceptibility/intermediate susceptibility at KEM (Pune) and ICH (Kolkata) were 84% and 66%, respectively, whereas it was 30% at SGPGI (Lucknow). The rates of susceptibility/intermediate susceptibility to both cefepime and cefotaxime were the highest at KEM (Pune) (69% and 41%, respectively), followed by ICH (Kolkata) (35% and 31%, respectively) and SGPI (Lucknow) (18% and 32%, respectively) (Figure 1).

**Figure 2:**
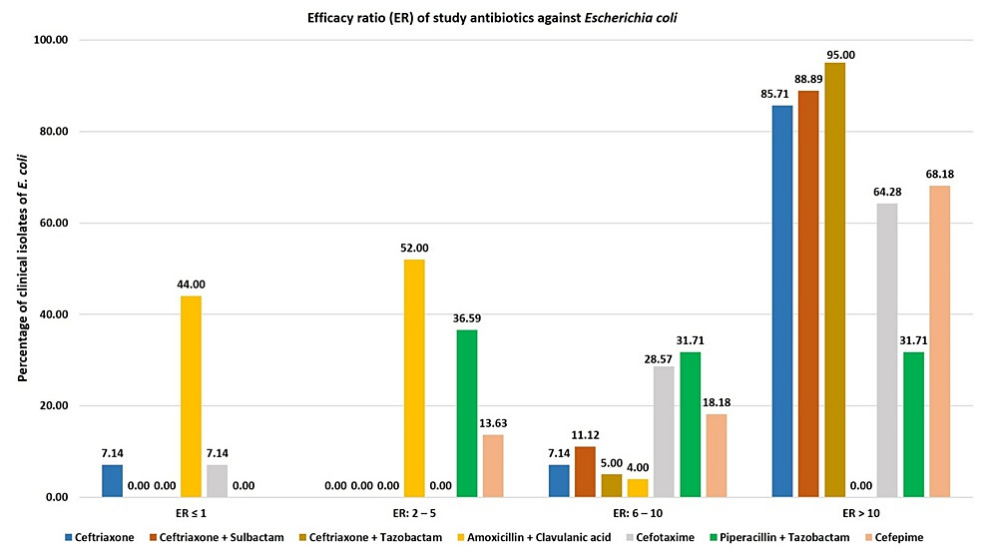
Efficacy ratio (ER) of study antibiotics against Escherichia coli.

The concentration ranges of susceptibility, intermediate susceptibility, and resistance obtained for the studied antibiotics against the predominant isolates (*E. coli *and *K. pneumoniae*) are presented in Table 1 along with the reference ranges. We performed ER analysis for these isolates. As shown in Table 2 and Figure 2, ceftriaxone alone and ceftriaxone in combination with sulbactam and tazobactam had ER >10 against 85%-95% of *E. coli* isolates, whereas comparator antibiotics had ER >10 against 31%-68% of *E. coli* isolates. The percentages of *E. coli* isolates with ER >10 in the case of ceftriaxone-based antibiotics were significantly higher than the corresponding percentages obtained for piperacillin + tazobactam and cefotaxime. Comparison with cefepime yielded statistical significance in the case of ceftriaxone + tazobactam (Table 3).

**Table 1:** Susceptibility ranges of study antibiotics against Escherichia coli and Klebsiella pneumoniae. All concentrations are in units of µg/mL. I: intermediate; MIC: minimum inhibitory concentration; R: resistant; S: susceptible

Bacteria			Susceptibility ranges						
			Ceftriaxone	Ceftriaxone + sulbactam	Ceftriaxone + tazobactam	Amoxicillin + clavulanic acid	Cefotaxime	Piperacillin + tazobactam	Cefepime
*Escherichia coli* (n = 70)	Current study	S (% of isolates)	0.032–0.75 (20.00%)	0.023–0.125 (25.71%)	0.023–0.125 (28.57%)	1–8 (35.71%)	0.023–1 (18.57%)	0.125–8 (47.14%)	0.064–4 (25.71%)
		I (% of isolates)	Nil	1.5–2 (5.71%)	1.5–3 (10.00s%)	12–16 (5.71%)	3 (2.85%)	24–≥32 (5.71%)	12–16 (5.71%)
		R (% of isolates)	>4 (80.00%)	≥4 (68.57%)	>4 (61.42%)	≥128 (58.57%)	>4 (54.28%)	>256 (22.85%)	>256 (44.28%)
	Reference	S	<1	≤1	<1	<8	<1	<16	<8
		I	2	2	2	-	2	32-64	16
		R	>4	≥4	>4	>32	>4	>128	>32
*Klebsiella pneumonia* (n = 37)	Current study	S (% of isolates)	0.064–0.125 (24.32%)	0.047–0.125 (18.91%)	0.032–0.094 (24.32%)	1–4 (21.62%)	0.047–0.125 (18.91%)	0.25–8 (37.83%)	≤2 (27.02%)
		I (% of isolates)	Nil	1.5 (1.42%)	2 (2.70%)	16 (5.40%)	3 (2.70%)	24-64 (10.81%)	16 (8.10%)
		R (% of isolates)	≥16 (75.67%)	≥6 (78.37%)	≥6 (72.97%)	>32 (72.97%)	>16 (78.37%)	>256 (51.35%)	>64 (64.86%)
	Reference	S	<1	≤1	<1	<8	<1	<16	<8
		I	2	2	2	-	2	32–64	16
		R	>4	≥4	>4	>32	>4	>128	>32

**Table 2:** Efficacy ratio of study antibiotics against Escherichia coli. n (%): number and percentage of isolates; ER: efficacy ratio; MIC: minimum inhibitory concentration Efficacy ratio (ER) = susceptible MIC breakpoint/MIC of clinical isolate Number of *E. coli* isolates = 70

Antibiotics	Susceptible, n (%)	Intermediate, n (%)	Resistant, n (%)	ER			
				ER ≤1, n (%)	ER: 2–5, n (%)	ER 6–10, n (%)	ER >10, n (%)
Ceftriaxone	14 (20.00%)	-	56 (80.00%)	1 (7.14%)	-	1 (7.14%)	12 (85.71%)
Ceftriaxone + sulbactam	18 (25.71%)	4 (5.71%)	48 (68.57%)	-	-	2 (11.12%)	16 (88.89%)
Ceftriaxone + tazobactam	20 (27.78%)	3 (4.28%)	47 (67.14%)	-	-	1 (5.00%)	19 (95.00%)
Amoxicillin + clavulanic acid	25 (35.71%)	4 (5.71%)	41 (58.57%)	11 (44.00%)	13 (52.00%)	1 (4.00%)	-
Cefotaxime	14 (20.00%)	-	56 (80.00%)	1 (7.14%)	-	4 (28.57%)	9 (64.28%)
Piperacillin + tazobactam	41 (58.57%)	9 (12.85%)	20 (28.57%)	-	15 (36.59%)	13 (31.71%)	13 (31.71%)
Cefepime	22 (31.43%)	7 (1.00%)	41 (58.57%)	-	3 (13.63%)	4 (18.18%)	15 (68.18%)

**Table 3:** Comparison of efficacy ratio (ER) of test antibiotics to other antibiotics against Escherichia coli (p-values). Note: The result of the statistical test carried out between the percentage of isolates for the antibiotics mentioned in the first column when compared to the antibiotics in the top row is given as a p-value in the cell at the intersection. The antibiotic comparison with no p-value either does not have susceptible samples or does not belong to the efficacy ratio range mentioned. P-values were not calculated for the ER range of 2-5 as none of the test antibiotics had an ER in this range.

ER	Ceftriaxone	Ceftriaxone + sulbactam	Ceftriaxone + tazobactam	Amoxicillin + clavulanic acid	Cefotaxime	Piperacillin + tazobactam	Cefepime
ER ≤1, n (%)							
Ceftriaxone	x	-	-	0.008	0.500	-	-
Ceftriaxone + sulbactam	-	x	-	-	-	-	-
Ceftriaxone + tazobactam	-	-	x	-	-	-	-
ER 6–10, n (%)							
Ceftriaxone	x	0.351	0.397	0.333	0.069	0.034	0.176
Ceftriaxone + sulbactam	0.351	x	0.241	0.184	0.103	0.047	0.267
Ceftriaxone + tazobactam	0.397	0.241	x	0.436	0.028	0.009	0.093
ER >10, n (%)							
Ceftriaxone	x	0.393	0.173	-	0.095	<0.001	0.119
Ceftriaxone + sulbactam	0.393	x	0.241	-	0.047	<0.001	0.059
Ceftriaxone + tazobactam	0.173	0.241	x	-	0.010	<0.001	0.013

**Figure 3:**
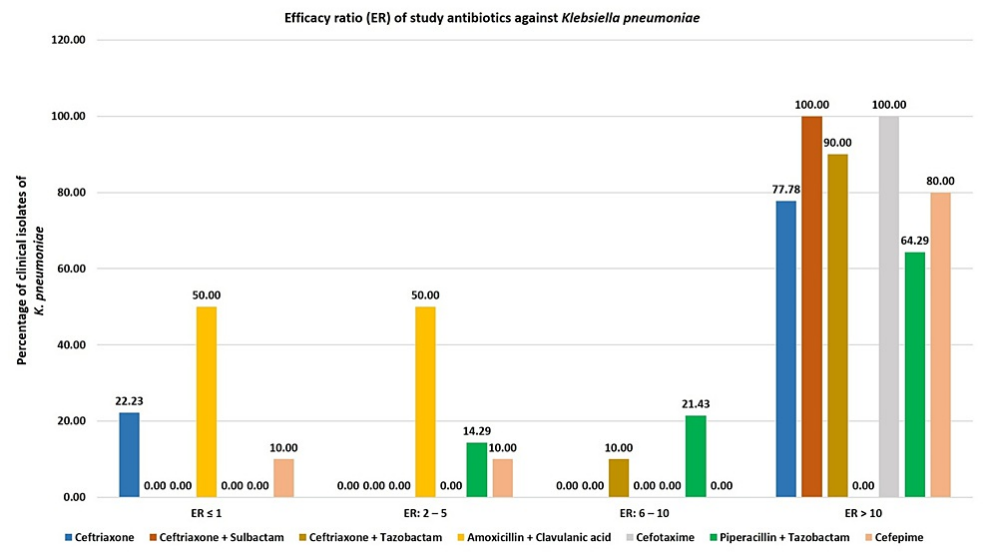
Efficacy ratio (ER) of study antibiotics against Klebsiella pneumoniae.

Analysis for* K. pneumoniae* isolates showed that ceftriaxone, ceftriaxone + sulbactam, and ceftriaxone + tazobactam had ER >10 against 78%, 100%, and 90% of isolates, respectively. Comparator antibiotics cefotaxime, piperacillin + tazobactam, and cefepime had ER >10 against 100%, 64%, and 80% of isolates, respectively (Table 4 and Figure 3).

**Table 4:** Efficacy ratio (ER) of study antibiotics against Klebsiella pneumoniae. n (%): number and percentage of isolates; ER: efficacy ratio; MIC: minimum inhibitory concentration Efficacy ratio (ER) = susceptible MIC breakpoint/MIC of clinical isolate Number of *K. pneumoniae* isolates = 37

Antibiotics	Susceptible, n (%)	Intermediate, n (%)	Resistant, n (%)	ER			
				ER ≤1, n (%)	ER: 2–5, n (%)	ER 6–10, n (%)	ER >10, n (%)
Ceftriaxone	9 (24.32%)	1 (2.70%)	27 (72.97%)	2 (22.23%)	-	-	7 (77.78%)
Ceftriaxone + sulbactam	7 (18.92%)	1 (2.70%)	29 (78.38%)	-	-	-	7 (100.00%)
Ceftriaxone + tazobactam	10 (27.02%)	-	27 (92.97%)	-	-	1 (10.00%)	9 (90.00%)
Amoxicillin + clavulanic acid	8 (21.62%)	2 (5.41%)	27 (72.97%)	4 (50.00%)	4 (50.00%)	-	-
Cefotaxime	7 (18.92%)	1 (2.70%)	29 (78.37%)	-	-	-	7 (100.00%)
Piperacillin + tazobactam	14 (37.84%)	4 (10.81%)	19 (51.35%)	-	2 (14.29%)	3 (21.43%)	9 (64.29%)
Cefepime	10 (27.03%)	3 (8.11%)	24 (64.86%)	1 (10.00%)	1 (10.00%)	-	8 (80.00%)

The percentages of *K. pneumoniae* isolates with ER >10 in the case of ceftriaxone-based antibiotics were significantly higher than the corresponding percentages obtained for piperacillin + tazobactam (Table 5).

**Table 5:** Comparison of efficacy ratio (ER) of test antibiotics to other antibiotics against Klebsiella pneumoniae (p-values). Note: The result of the statistical test carried out between the percentage of isolates for the antibiotics mentioned in the first column when compared to the antibiotics in the top row is given as a p-value in the cell at the intersection. The antibiotic comparison with no p-value either does not have susceptible samples or does not belong to the efficacy ratio range mentioned. P-values were not calculated for the ER range of 2-5 as none of the test antibiotics had an ER in this range.

ER	Ceftriaxone	Ceftriaxone + sulbactam	Ceftriaxone + tazobactam	Amoxicillin + clavulanic acid	Cefotaxime	Piperacillin + tazobactam	Cefepime
ER ≤1, n (%)							
Ceftriaxone	x	-	-	0.115	-	-	0.232
Ceftriaxone + sulbactam	-	x	-	-	-	-	-
Ceftriaxone + tazobactam	-	-	x	-	-	-	-
ER 6–10, n (%)							
Ceftriaxone	x	-	-	-	-	-	-
Ceftriaxone + sulbactam	-	x	-	-	-	-	-
Ceftriaxone + tazobactam	-	-	x	-	-	0.229	-
ER > 10, n (%)							
Ceftriaxone	x	0.091	0.232	-	0.091	0.245	0.452
Ceftriaxone + sulbactam	0.091	x	0.194	-	0.999	0.035	0.103
Ceftriaxone + tazobactam	0.232	0.194	x	-	0.194	0.076	0.264

Analysis for other isolates revealed that 100% of *S. typhi* and *S. enterica* (number of isolates = six) were susceptible to all study antibiotics. Of the isolated *P. aeruginosa* (number of isolates = 30), 71% showed susceptibility/intermediate susceptibility to ceftriaxone. Among the *P. mirabilis* (number of isolates = four), susceptibility/intermediate susceptibility was displayed by 100% of isolates to ceftriaxone and ceftriaxone + tazobactam and by 75% of isolates to ceftriaxone + sulbactam. Of the isolated gram-positive *S. aureus* (number of isolates = 19), 47% showed susceptibility/intermediate susceptibility to both ceftriaxone + sulbactam and ceftriaxone + tazobactam.

Of the 180 isolates, 90 (50%) were ESBL-producing bacteria and 69 (38.33%) were MBL-producing bacteria, all of which were gram-negative. These included 52 (29%) isolates that produced both ESBL and MBL. Among the 70 isolates of *E. coli*, 26 (37.14%) were ESBL producers, nine (12.86%) were MBL producers, and 14 (20.00%) produced both ESBL and MBL. Among the 37 isolates of *K. pneumoniae*, nine (24.32%) were ESBL producers, five (13.51%) were MBL producers, and 16 (43.24%) produced both ESBL and MBL.

Analysis showed that of the 40 ESBL-producing *E. coli *isolates, all those susceptible to ceftriaxone + sulbactam (four isolates), ceftriaxone + tazobactam (six isolates), and cefepime (five isolates) had ER >10. The percentage of isolates with ER >10 in the case of ceftriaxone in combination with BLIs was significantly higher than the corresponding percentage obtained for piperacillin + tazobactam. All 23 MBL-producing *E. coli* isolates were resistant to the test antibiotics (Tables 6, 7).

**Table 6:** Efficacy ratio (ER) of study antibiotics against ESBL- and MBL-producing Escherichia coli. n (%): number and percentage of isolates; ER: efficacy ratio; ESBL: extended-spectrum beta-lactamase; MSL: metallo-beta-lactamase; MIC: minimum inhibitory concentration Efficacy ratio (ER) = susceptible MIC breakpoint/MIC of clinical isolate Number of ESBL-producing *E. coli* isolates = 40. Number of MBL-producing *E. coli* isolates = 23

Antibiotics	Susceptible, n (%)	Intermediate, n (%)	Resistant, n (%)	ER			
				ER ≤1, n (%)	ER: 2–5, n (%)	ER 6–10, n (%)	ER >10, n (%)
ESBL-producing *E. coli*							
Ceftriaxone	-	-	40 (100.00%)	-	-	-	-
Ceftriaxone + sulbactam	4 (10.00%)	1 (2.50%)	35 (87.50%)	-	-	-	4 (100.00%)
Ceftriaxone + tazobactam	6 (15.00%)	3 (7.50%)	31 (77.50%)	-	-	-	6 (100.00%)
Amoxicillin + clavulanic acid	8 (20.00%)	1 (2.50%)	31 (77.50%)	6 (75.00%)	2 (25.00%)	-	-
Cefotaxime	-	-	40 (100%)	-	-	-	-
Piperacillin + tazobactam	19 (47.50%)	7 (17.50%)	14 (35.00%)	-	10 (52.63%)	1 (5.26%)	8 (42.10%)
Cefepime	5 (12.50%)	4 (10.00%)	31 (77.50%)	-	-	-	5 (100.00%)
MBL-producing *E. coli*							
Ceftriaxone	-	-	23 (100.00%)	-	-	-	-
Ceftriaxone + sulbactam	-	-	23 (100.00%)	-	-	-	-
Ceftriaxone + tazobactam	-	-	23 (100.00%)	-	-	-	-
Amoxicillin + clavulanic acid	1 (4.35%)	1 (4.35%)	21 (91.30%)	1 (100.00%)	-	-	-
Cefotaxime			23 (100.00%)	-	-	-	-
Piperacillin + tazobactam	1 (4.35%)	3 (13.04%)	19 (82.61%)	-	-	1 (100.00%)	-
Cefepime	-	1 (4.35%)	22 (95.65%)	-	-	-	-

**Table 7:** Comparison of efficacy ratio (ER) of test antibiotics to other antibiotics against ESBL-producing Escherichia coli (p-values). Note: The result of the statistical test carried out between the percentage of isolates for the antibiotics mentioned in the first column when compared to the antibiotics in the top row is given as a p-value in the cell at the intersection. The antibiotic comparison with no p-value either does not have susceptible samples or does not belong to the efficacy ratio range mentioned. P-values were not calculated for ER ranges apart from ER >10 as none of the test antibiotics had an ER in this range.

ER	Ceftriaxone	Ceftriaxone + sulbactam	Ceftriaxone + tazobactam	Amoxicillin + clavulanic acid	Cefotaxime	Piperacillin + tazobactam	Cefepime
ER >10, n (%)							
Ceftriaxone	x	-	-	-	-	-	-
Ceftriaxone + sulbactam	-	x	-	-	-	0.034	0.999
Ceftriaxone + tazobactam	-	-	x	-	-	0.012	0.999

ER analysis further showed that among the *K. pneumoniae* isolates, the three ESBL producers and one MBL producer susceptible to ceftriaxone + tazobactam had ER >10. Differences with comparator antibiotics were not statistically significant. Of the 25 ESBL-producing *K. pneumoniae* isolates, susceptibility to ceftriaxone and ceftriaxone + sulbactam was displayed by one isolate in each case, with ER in the range of 6-10. The only comparator antibiotic with a similar ER (for one susceptible isolate) was amoxicillin + clavulanic acid. Statistical analysis for comparison between test and comparator antibiotics was not performed as ER was based on single isolates in all three cases. All 21 MBL-producing *K. pneumoniae* isolates were resistant to ceftriaxone and ceftriaxone + sulbactam (Table 8).

**Table 8:** Efficacy ratio (ER) of antibiotics against ESBL- and MBL-producing Klebsiella pneumoniae. n (%): number and percentage of isolates; ER: efficacy ratio; ESBL: extended-spectrum beta-lactamase; MSL: metallo-beta-lactamase; MIC: minimum inhibitory concentration Efficacy ratio (ER) = susceptible MIC breakpoint/MIC of clinical isolate Number of ESBL-producing *K. pneumoniae* isolates = 25. Number of MBL-producing *K. pneumoniae* isolates = 21

Antibiotics	Susceptible, n (%)	Intermediate, n (%)	Resistant, n (%)	ER			
				ER ≤1, n (%)	ER: 2–5, n (%)	ER 6–10, n (%)	ER >10, n (%)
ESBL-producing *K. pneumoniae*							
Ceftriaxone	1 (4.00%)	-	24 (96.00%)	-	-	1 (100.00%)	-
Ceftriaxone + sulbactam	1 (4.00%)	1 (4.00%)	23 (92.00%)	-	-	1 (100.00%)	-
Ceftriaxone + tazobactam	3 (12.00%)	1 (4.00%)	21 (84.00%)	-	-	-	3 (100.00%)
Amoxicillin + clavulanic acid	2 (8.00%)	2 (8.00%)	21 (84.00%)	-	1 (50.00%)	1 (50.00%)	-
Cefotaxime	1 (4.00%)	1 (4.00%)	23 (92.00%)	-	-	-	1 (100.00%)
Piperacillin + tazobactam	7 (28.00%)	3 (12.00%)	15 (60.00%)	1 (14.29%)	2 (28.57%)	-	4 (57.14%)
Cefepime	3 (12.00%)	3 (12.00%)	19 (76.00%)	-	-	-	3 (100.00%)
MBL-producing *K. pneumoniae*							
Ceftriaxone	-	-	21 (100.00%)	-	-	-	-
Ceftriaxone + sulbactam	-	-	21 (100.00%)	-	-	-	-
Ceftriaxone + tazobactam	1 (4.76%)	-	20 (95.24%)	-	-	-	1 (100.00%)
Amoxicillin + clavulanic acid	-	-	21 (100.00%)	-	-	-	-
Cefotaxime	-	-	21 (100.00%)	-	-	-	1 (100.00%)
Piperacillin + tazobactam	1 (4.76%)	4 (19.05%)	16 (76.19%)	-	-	-	1 (100.00%)
Cefepime	-	2 (9.52%)	19 (90.48%)	-	-	-	-

## Discussion

The β-lactam antibiotics are the largest and currently the most widely used antibacterial agents. However, the susceptibility of infectious organisms to third-generation cephalosporins has decreased over the years due to the increasing prevalence of drug-resistant organisms which might be attributed to the excessive use of broad-spectrum antibiotics. This, in turn, is responsible for difficult-to-treat hospital-associated infectious diseases as causative organisms develop AMR.

A well-characterized mechanism of resistance includes the production of β-lactamases that hydrolyze and deactivate several antibiotics containing β-lactam ring. To overcome bacterial resistance due to β-lactamase production, these antibiotics are often combined with BLIs such as clavulanic acid, sulbactam, and tazobactam. In this study, we evaluated the MIC and susceptibility of clinical isolates to ceftriaxone alone and ceftriaxone in combination with sulbactam and tazobactam. We further compared the ERs of these antibiotics to corresponding values of other broad-spectrum β-lactam antibiotics, namely, amoxicillin + clavulanic acid, piperacillin + tazobactam, cefotaxime, and cefepime.

Of the 180 clinical isolates obtained from three study sites, 89% were gram-negative and 11% were gram-positive. Among the gram-negative isolates, 43%, 35%, and 33% were susceptible/intermediately susceptible to ceftriaxone at KEM (Pune), SGPGI (Lucknow), and ICH (Kolkata), respectively. The highest rate of susceptibility/intermediate susceptibility to ceftriaxone was observed at KEM (Pune). The rate of susceptibility/intermediate susceptibility to ceftriaxone at SGPGI (Lucknow) was higher than the corresponding values obtained for all other antibiotics tested at this site. Site-specific differences observed in this study could be related to varying antimicrobial policies at different sites.

Susceptibility/intermediate susceptibility rates of ceftriaxone-based antibiotics against *E. coli *and *K. pneumoniae* were in the range of 20%-29% and 19%-24%, respectively; these values were comparable to a few of the comparator antibiotics. All three ceftriaxone-based antibiotics showed susceptibility/intermediate susceptibility rates of 100% against *S. typhi *and *S. enterica*. Furthermore, 100% of *P. mirabilis* and 71% of *P. aeruginosa* isolates showed susceptibility/intermediate susceptibility to ceftriaxone. Also, 75% of *P. mirabilis *and 47% of *S. aureus *showed susceptibility/intermediate susceptibility to ceftriaxone + sulbactam. The susceptibility rates of ceftriaxone + tazobactam against *P. mirabilis *and *S. aureus* were 100% and 47%, respectively.

A recent study from India showed that 11.6% of *Salmonella *spp. isolates from enteric fever patients were resistant to ceftriaxone [8]. We report a 100% susceptibility of *Salmonella *spp. isolates from enteric fever to ceftriaxone; however, we are limited by the small sample size (number of isolates = six). In a study conducted in Pakistan, 96.1% of *S. aureus*, 95% o*f E. coli*, 92.7% of *P. aeruginosa*, 89.4% of *K. pneumoniae*, 87.2% of *S. typhi*, and 83.8% of *P. mirabilis* isolates showed susceptibility toward ceftriaxone [9]. This is aligned with our study showing a 100% susceptibility rate of *S. typhi* to ceftriaxone. However, the susceptibility of other bacterial isolates to ceftriaxone was not comparable. Barring the consideration that the earlier study was from a different geographical area, it is possible that resistance to ceftriaxone has crept in as it has been over a decade since the publication of this study. Prakash et al. in 2005 reported that 80.9% and 6.4% of *E. coli* isolates and 70.8% and 4.2% of *K. pneumoniae* isolates were resistant to ceftriaxone and ceftriaxone + tazobactam, respectively [10]. Our study revealed comparable results with resistance noted in 80% of *E. coli* and 76% of *K. pneumoniae* isolates. However, a higher resistance rate was noted against ceftriaxone + tazobactam plausibly related to enhanced AMR over the years.

ER plays a crucial role in establishing the hierarchy of preference in a clinical setting, both within and across various classes of antimicrobials, and making informed decisions regarding the optimal selection of antibiotics for the treatment of specific infections. A drug with a higher ER is expected to be more effective compared to a drug with a lower ER [7]. We compared the antibiotics based on their ERs against *E. coli *and *K. pneumoniae*, the two predominant clinical isolates in our study. Our results showed that the ERs of ceftriaxone-based antibiotics against most isolates of *E. coli *and *K. pneumoniae* were greater than 10, implying that the observed MIC for susceptibility is 10-fold lower than the susceptibility breakpoint. This, in other words, the antibiotic is expected to be effective against the susceptible bacteria at a lower concentration. Moreover, the degree of susceptibility of *E. coli *to ceftriaxone-based antibiotics was higher than that of all comparator antibiotics. Differences between the test and comparator antibiotics were statistically significant for multiple instances. In the case of *K. pneumoniae*, the efficacy of ceftriaxone-based antibiotics was better than or comparable to all other antibiotics tested.

Furthermore, all ESBL-producing *E. coli* isolates susceptible to ceftriaxone + sulbactam and ceftriaxone + tazobactam had ER >10. All ESBL- and MBL-producing isolates of *K. pneumoniae* susceptible to ceftriaxone + tazobactam also had ER >10. ESBL-producing *K. pneumoniae* isolates susceptible to ceftriaxone and ceftriaxone + sulbactam had ER in the range of 6-10. However, all MBL-producing *E. coli *isolates were resistant to ceftriaxone-based antibiotics, and all MBL-producing *K. pneumoniae* isolates were resistant to ceftriaxone and ceftriaxone + sulbactam. Thus, ceftriaxone + tazobactam holds promise against ESBL-producing variants of these bacterial species. However, as the number of variants in the current study was limited, further investigations are required to establish this.

Earlier studies have proven the efficacy and safety of ceftriaxone in patients. With salient features such as a long elimination half-life, a convenient dosing schedule, and a good tolerability profile [11,12], ceftriaxone is a promising antibiotic. Cumulatively, our results highlight the efficacy of ceftriaxone and ceftriaxone in combination with sulbactam and tazobactam in clinical isolates, particularly *E. coli *and* K. pneumoniae*. A practical implication of our findings would be to guide treatment strategies in infectious diseases caused by these organisms. Clinicians might find it appropriate to prescribe ceftriaxone or its combinations as a first-line therapy or to patients where the causative organism is known and the patients are resistant to other antibiotics.

A limitation of the current study is the small number of clinical samples investigated, and the small number of samples that represent MBL-producing strains and bacterial species apart from *E. coli *and *K. pneumoniae*. These might limit the generalizability of the findings of this study. Nevertheless, based on our initial findings, ceftriaxone-based antibiotics appear to be a promising avenue to explore. Future studies with a larger number of samples are required to ascertain with confidence the results of susceptibility testing using ceftriaxone-based antibiotics and to investigate their efficacy in variants and bacterial species that are not predominant. We have used the breakpoint of ceftriaxone as a proxy for ceftriaxone + sulbactam and ceftriaxone + tazobactam due to the absence of specific breakpoints for these combinations. We acknowledge that this might introduce some uncertainty in the interpretation of the susceptibility data presented here, but we hope that our data will contribute to the formulation of breakpoints for these antibiotics and aid clinicians in the future.

## Conclusions

This study provides convincing results to support the efficacy of ceftriaxone alone and ceftriaxone in combination with sulbactam and tazobactam against prevalent infectious organisms such as *E. coli* and *K. pneumoniae*. Our study also supports that the comparison of ERs is a better measure than the direct comparison of susceptibility rates and MIC values. Depending on our study results, we believe that robust data on the susceptibility of various bacterial species to commonly used antibiotics would guide treatment decisions for optimum patient management based on the identification of the causative organism. In addition to formulating stringent antibiotic policy guidelines, regular surveillance programs should be conducted to assess the sensitivity and susceptibility of bacteria against commonly prescribed antibiotics. Appropriate use of antibacterial agents must be highly emphasized to minimize the development of AMR. Concerted efforts are essential to overcome AMR and ensure that mankind continues to benefit from the use of antibiotics.
